# Treatment of Chronic Hepatitis C Can Improve Glycemic Control in Patients with Type 2 Diabetes

**DOI:** 10.1155/2018/5260510

**Published:** 2018-09-17

**Authors:** Nalan Okuroglu, Meltem Sertbas, Ali Ozdemir

**Affiliations:** University of Health Sciences, Fatih Sultan Mehmet Training and Research Hospital, Department of Internal Medicine, Istanbul, Turkey

## Abstract

A 73-year-old female patient with a history of type 2 DM for seven years was admitted to our out-patient clinic with a complaint of frequent hypoglycemic episodes. She was receiving basal- bolus insulin treatment. She underwent liver transplantation 20 months ago due to end stage liver disease caused by HCV infection genotype 1b. While she was still on tacrolimus for liver transplantation, she received direct acting antiviral agents including fix dose ledipasvir-sofosbuvir with ribavirin. Biochemical analysis showed fasting plasma glucose of 105 mg/dl and postprandial glucose of 200 mg/dl, glycosylated hemoglobin A1c of 4.8%, and c-peptide of 3.17 ng/ml. After achieving successfully virologic response with antiviral therapy, the patient stayed euglycemic and was no longer in need to any medication including insulin and the patient was followed only by dietary regulation. Achievement of the virological response in treatment of HCV infection can improve not only the liver status, but also the extrahepatic manifestations including type 2 DM.

## 1. Introduction

Hepatitis C virus (HCV) infection which has a prevalence of 2.8% is one of the major causes of hepatitis, cirrhosis, and hepatocellular carcinoma [1]. Several studies have reported that the prevalence of diabetes mellitus (DM) is higher among HCV-associated hepatitis compared to other viral hepatitis. The underlying mechanisms of DM development can be listed as HCV-associated glucose intolerance, insulin resistance (IR), increased inflammatory response, liver fibrosis, and the direct effect of HCV on insulin signaling. Recently, a few clinical trials and cases showed improvement of the regulation of DM after antiviral treatment. We report a case with type 2 DM who had liver transplantation due to chronic HCV infection 20 months ago and undergoing basal-bolus insulin therapy already. After achieving successfully virologic response with antiviral therapy the patient stayed euglycemic and was no longer in need to any medication including insulin.

## 2. Case Presentation

A 73-year-old female patient with type 2 DM was referred to our clinic for low sugar episodes. She had been receiving insulin therapy for 7 years. In medical history liver transplantation was performed 20 months ago due to end stage liver disease caused by HCV infection genotype 1b. She had a body mass index (BMI) of 31.9 kg/m^2^ and was using intensive subcutaneous insulin injections four times daily: insulin glargine 24 U at bedtime and insulin aspart 16U three times a day before each meal. While she was still on tacrolimus 2.5 mg and mycophenolate mofetil 1000 mg daily, after liver transplantation for nearly 20 months, she received direct acting antiviral agents (DAAs) including fix dose combination ledipasvir-sofosbuvir (90 mg-400 mg) plus ribavirin, for 24 weeks, and achieved virologic response. After DAAs and ribavirin treatment was completed, she began to experience severe hypoglycemia and therefore the insulin aspart was stopped. Since hypoglycemia persists, she also discontinued insulin glargine 1 week ago. Her vital signs were normal and physical examination was unremarkable. Biochemical analysis showed fasting plasma glucose (FG) of 105 mg/dl and postprandial glucose of 200 mg/dl. Surprisingly, glycosylated hemoglobin A1c (HbA1c) was 4.8% and c-peptide was 3.17 ng/ml. Liver and renal function test results were in the normal reference range. Home blood glucose measurements also showed a normal course of glucose and the patient was followed only by dietary regulation.

## 3. Discussion

The development of diabetes in HCV infection is well known and is called "hepatogenos diabetes". The main reason for the pathogenesis of "hepatogenous diabetes" is especially IR associated with impaired insulin secretion and sensitivity. Also, IR has been shown to increase liver fibrosis, reduce antiviral treatment response, and increase hepatocellular carcinoma (HCC). IR and hepatic steatosis are the characteristic features of HCV. IR was an independent risk factor in the development of fibrosis in patients with chronic HCV infection [2]. There are two types of IR: metabolic and viral. Compared to other inflammatory liver diseases, hepatosteatosis was found to be more common in chronic hepatitis C virus infection. The direct effect of hepatitis C virus core protein induces fat accumulation in hepatocytes and causes hepatic steatosis. Both fat accumulation and hepatosteatosis have been related to elevations of serum aminotransferase levels in HCV infections rather than DM. It has been reported that nonstructural protein 5A (NS5A) encoded by the HCV RNA genome is involved in insulin receptor substrate-1 (IRS-1) phosphorylation and regulates the insulin signaling pathway [3]. In addition, NS5A transfected cells, decreased the levels of serin phosphorylation that facilitate gluconeogenesis, and inhibited the synthesis of glycogen.

Moreover, Massini et al. showed that HCV can affect pancreatic *β*-cells and induce morphological cell changes [4]. In another study Wang suggested that HCV infection may cause pancreatic beta cells death by an endoplasmic reticulum (ER) stress-involved effect [5].

By the clinical use of novel DAAs including protease inhibitors, and nucleotide and non-nucleotide polymerase inhibitors like NS5a-Ns5b inhibitors, with or without ribavirin, high HCV clearance rates have been achieved. There are clinical case reports showing that FG and HbA1c recover after HCV eradication has been achieved [6, 7]. It has been suggested that coexisting diabetes or hyperlipidemia might interact with the response to DAAs and patients with blood glucose <126 mg/dl had a higher virologic response [8].

In our case, high c-peptide levels and withdrawal of the insulin with achieving virologic response support the idea that not only liver but also pancreas tissue and *β*-cells may be infected by HCV. Another question about the case is the liver transplantation that had been performed 20 months ago. Liver transplantation is an effective treatment in advance liver diseases. It is well known that transplant recipients have a higher risk of new onset diabetes especially under immunosuppressive treatments [9]. While the patient was still on tacrolimus 2.5 mg and mycophenolate mofetil 1000 mg daily, the need for insulin therapy continued after the liver transplantation. The improvement of glycemic regulation after permanent viral response, but not liver transplantation, suggests the evidence that pancreatic *β*-cells take place in extrahepatic manifestations of chronic HCV infection.

## 4. Conclusion

Achievement of the virological response in treatment of HCV infection can improve not only the liver status, but also the extrahepatic manifestations including type 2 DM. Thus, hypoglycemia should be kept in mind in diabetic patients treated by DAAs with high virological response.
